# Serious Game Design and Clinical Improvement in Physical Rehabilitation: Systematic Review

**DOI:** 10.2196/20066

**Published:** 2021-09-23

**Authors:** Catarina Vieira, Carla Ferreira da Silva Pais-Vieira, João Novais, André Perrotta

**Affiliations:** 1 Research Center for Science and Technology of the Arts Universidade Católica Portuguesa Porto Portugal; 2 Centro de Interdisciplinar de Investigação em Saúde Universidade Católica Portuguesa Porto Portugal; 3 Católica Porto Business School Universidade Católica Portuguesa Porto Portugal; 4 Research Centre for Informatics and Systems Informatics Engineering Department Universidade de Coimbra Coimbra Portugal

**Keywords:** serious games, physical rehabilitation, systematic review, physical impairment, game design, game characteristics, stroke, multiple sclerosis, cerebral palsy

## Abstract

**Background:**

Serious video games have now been used and assessed in clinical protocols, with several studies reporting patient improvement and engagement with this type of therapy. Even though some literature reviews have approached this topic from a game perspective and presented a broad overview of the types of video games that have been used in this context, there is still a need to better understand how different game characteristics and development strategies might impact and relate to clinical outcomes.

**Objective:**

This review assessed the relationship between the characteristics of serious games (SGs) and their relationship with the clinical outcomes of studies that use this type of therapy in motor impairment rehabilitation of patients with stroke, multiple sclerosis, or cerebral palsy. The purpose was to take a closer look at video game design features described in the literature (game genre [GG], game nature [GN], and game development strategy [GDS]) and assess how they may contribute toward improving health outcomes. Additionally, this review attempted to bring together medical and game development perspectives to facilitate communication between clinicians and game developers, therefore easing the process of choosing the video games to be used for physical rehabilitation.

**Methods:**

We analyzed the main features of SG design to obtain significant clinical outcomes when applied to physical rehabilitation of patients recovering from motor impairments resulting from stroke, multiple sclerosis, and cerebral palsy. We implemented a PRISMA (Preferred Reporting Items for Systematic Reviews and Meta-Analyses) database-adjusted electronic search strategy for the PubMed, IEEE Xplore, and Cochrane databases.

**Results:**

We screened 623 related papers from 2010-2021 and identified 12 that presented results compatible with our inclusion criteria. A total of 512 participants with stroke (8 studies, 417 participants), cerebral palsy (1 study, 8 participants), and multiple sclerosis (2 studies, 46 participants) were included; 1 study targeting the elderly (41 participants) was also included. All studies assessed motor, sensory, and functional functions, while some also measured general health outcomes. Interventions with games were used for upper-limb motor rehabilitation. Of the 12 studies, 8 presented significant improvements in at least one clinical measurement, of which 6 presented games from the casual GG, 1 combined the casual, simulation, and exergaming GGs, and 2 combined the sports and simulation GGs.

**Conclusions:**

Of the possible combinations of game design features (GG, GN, and GDS) described, custom-made casual games that resort to the first-person perspective, do not feature a visible player character, are played in single-player mode, and use nonimmersive virtual reality attain the best results in terms of positive clinical outcomes. In addition, the use of custom-made games versus commercial off-the-shelf games tends to give better clinical results, although the latter are perceived as more motivating and engaging.

## Introduction

### Background

The concept of serious games (SGs) emerged with Abt in 1970. The author described SGs as games that “have an explicit and carefully thought-out educational purpose and are not intended to be played primarily for amusement,” therefore referring to an innovative approach to education in its various forms [1]. Initially referring to board and card games, the proposed concept endured, adapting itself to the technological advances taking place worldwide, ensuring that it is still applicable in the computer age that characterizes the twenty-first century [2]. In parallel, SG applications emerged into other fields that embodied other objectives and purposes [3]. For example, SGs have been used for a wide range of health care purposes: from education of medical personnel to health monitoring, health management, and rehabilitation [4]. Specifically, SGs in physical rehabilitation aim to provide an intervention context for reacquisition, recovery, or maintenance of the self’s physical faculties (ie, motor and sensorial functions) to ensure the quality of life (QoL) of patients. It usually encompasses post-stroke rehabilitation (both upper limb and lower limb), balance and gait training, orthopedic rehabilitation, and stimulation of physical activity for patients suffering from pathologies such as cerebral palsy, multiple sclerosis, or Parkinson [4-6]. Thus, the SG definition/applications grew broader, now referring to any video game that aims to convey some sort of message or input—be it knowledge, a skill, or something else—to the players, while preserving the characteristics that makes it be accepted and categorized as a video game [3,4].

On the design and development perspective, a video game—be it serious or not—can take several paths; therefore the array of variables that need to be considered is vast [7]. In this sense, game design elements, such as the game genre (GG), game nature (GN) [5,8,9], and game development strategy (GDS) are necessary to develop, deploy, and implement a video game. The GG encompasses sports, simulation, role-playing games, fighting, shooting, strategizing, etc [9]. Moreover, the design of a video game also refers to GN features [7], such as player perspective-taking (first-person perspective [1PP] vs third-person perspective [3PP]), game-play mode (multiplayer vs single player), type of scenery/in-game environment (realistic, fantasy-themed vs simple), the presence/absence of playable characters, and the level of immersion applied to the use of immersive or nonimmersive virtual reality (VR). From the GDS perspective, some studies have used custom-made SGs [10-17]—games that were tailored and developed specifically for the study in question—while others have opted for a direct-to-consumer approach by resorting to commercial off-the-shelf (COTS) titles [18-21], such as those available for the discontinued Nintendo Wii [22].

Past studies have tried to understand how game design elements can influence both game enjoyment and player engagement from different points of view: from a commercial/entertainment/general perspective [23-25], from a gamification perspective [26], or in terms of SGs targeting education [27]. One of the main and most studied applications of SGs in health care is the education and training of medical personnel [28]. Being 1 of the sole medical application areas for SGs that has been systematized in terms of, for example, the video GG, it has been demonstrated that for this purpose, the GG that is used the most is simulation, once it answers the specific needs of professional training [28,29]. Although the impact of simulation games for medical education has been quantified before by Cook et al [30] and Cheng et al [31], there is little to no research done (concerning outcome influence) for purposes other than education. In addition, Hookham and Nesbitt [29] analyzed several SGs used for education, including that of budding health care professionals. Their study proved that for these purposes, simulations and puzzles are the most common genres, since they are the video GGs that best suit the needs that the SG tries to cover [29]. However, although they have systematized information of this nature of SGs for education, to the best of our knowledge, there are no studies that analyze the game design elements (GG, GN, GDS) in games applied in the context of health care when the consumer is the patient instead of a professional in training. From the systematic content analysis of games for health presented by Lu and Kharazzi [8] as well as from the taxonomy proposed by Rego et al [5], we can get an overall picture of the use of these distinct genres in the context of health care. Lu and Kharazzi [8] present an overview of SGs developed specifically for health care between 1983 and 2016. Of the 1553 systematized games, 580 targeted cognitive training, while only 60 targeted physical activity and 5 addressed strokes. Of the analyzed games, the 3 predominant genres were puzzle, casual game, and simulation. Despite the thorough systematization of SGs applied to health care and their various aspects concerning the GG, GN and GDS, this review does not present any sort of conclusion that attempts to relate those aspects to clinical outcomes and/or patient improvement.

Altogether, even though several studies have adopted the SG approach to targeting physical rehabilitation, assessing the clinical efficacy and relevance of using video games [10-21], the relationship between intrinsic game design characteristics and clinical outcomes still needs to be further developed. This gap of knowledge has been previously pointed out, not only in the health care field, but also in relation to SGs and their many possible applications [32,33] Thus, there is a need to systematize studies that have achieved improvements by applying SGs for physical rehabilitation in order to find patterns and links between SG design (GG, GN, and GDS) and clinical improvement.

### Objectives

This systematic review aims to identify, evaluate, and summarize the features of SG design (GG, GN, and GDS) that significantly improve patient outcomes in physical rehabilitation. In this sense, we provided a qualitative synthesis that attempts to understand which video GG, GN features, and GDS may carry a link to attain significant clinical outcomes. To achieve our aim, we mapped both the characteristics of game design and the clinical outcomes. Specifically, we aimed to answer the question of what features of the game would be most relevant to improving clinical outcomes or may contribute to clinical intervention success.

## Methods

### Databases and Search Strategy

This systematic survey included papers written in English, published from 2010 to 2021, identified using the PubMed, IEEE Xplore, and Cochrane databases. We searched the titles, abstracts, and keywords of database entries using the following keywords: “serious games” AND “stroke” OR “cerebral palsy” OR “multiple sclerosis” OR “physical rehabilitation.” Search results from each database were merged and sorted for removal of duplicates. Afterward, titles and abstracts were screened according to their relevance to the research.

### Inclusion and Exclusion Criteria

The obtained results were screened according to the selection criteria (Textbox 1). Papers were included if they featured a clear description of game-based physical rehabilitation with specific descriptions of the game used in terms of its nature, featured the clinical test of the game on humans with control groups, and included quantitative results of performance measures. Non–peer-reviewed material, books, theses, and published posters were excluded. Additionally, studies were excluded if they (1) described the use of SGs for cognitive rehabilitation, (2) did not mention the design of the video game used for rehabilitation, (3) merely described prototypes without any actual clinical test, (4) had no quantitative results or had results assessed only qualitatively, (5) did not use control groups, or (6) did not have accessible full text.

**Selection criteria for systematic literature review.**

**Inclusion criteria**

- Papers that include game-based physical rehabilitation
- Papers that detail the aspects of the design of the applied video game
- Papers that detail the aspects of the design of the applied video game
- Papers that feature any sort of clinical test and present clear qualitative results of performance measures
- Other papers relevant to the research question

**Exclusion criteria**

- Papers that mention serious game (SG) design applied to cognitive rehabilitation
- Papers that mention an SG but do not specify the design of the game
- Papers that do not quantify the performance measures of the participants
- Papers that do not use control groups
- Books, theses, and published posters

### Data Extraction

We followed the PRISMA (Preferred Reporting Items for Systematic Reviews and Meta-Analyses) [34] guidelines for data extraction. The collected data comprised the study (authors, year of publication, study design, number of participants), sample information (gender, age, clinical information), game characteristics (ie, GN, GG, GDS), measures and clinical assessment information, and intervention characteristics (eg, number of rehabilitation sessions per week, session duration, total number of rehabilitation sessions). The primary outcome measure for this review referred to the clinical measurements (measurements, results, statistical significance), which were systematically extracted.

Given that the primary objective of this review was to access the relevant information regarding the relationship between game characteristics and the clinical outcomes of the respective studies, the selected papers were primarily organized and analyzed in terms of their GG, GN, and GDS aspects. The definition used for each game characteristics is presented in Table 1.

**Table 1:** Definition of each video GG^a^ found in this review [9].

Video games characteristics	Description
Health and wellness, fitness, exergaming	Video games that are also a form of exercise and that rely on technology that tracks body movement and (or) gestures.
Casual games	This category includes games that feature simple game play and objectives, including drag-and-drop games or point-and-click games.
Simulation	Games that aim to closely simulate aspects of a real or fictional reality. They seek to provide enjoyment through re-enactment.
Sports	Games that simulate the sporting experience. They focus on the experience of playing the sport or on the strategy behind the sport.
Game perspective (1PP^b^/3PP^c^)	The player camera angle/perspective. Games can be presented to the user either in 1PP or 3PP. In 1PP, the player experiences and interacts with the game through the playable character/avatar’s eyes, that is, the game action is observed the same way the user would experience the real world, which gives the player a sense of “being” the character (eg, *Overwatch*). In 3PP, the player is distanced from the game’s action by allowing them to control a playable character or avatar that they can see (eg, *New Super Mario Bros*) [35].
Game-play mode (single player/multiplayer)	Refers to whether the game is single-player (only one user can play the game at a time) or multiplayer (allows for different users to play the game at the same time and interact with each other) [36].
Presence of visible playable characters (yes/no)	Refers to whether the user has to control an avatar or a character in order to play the game. Playable characters are often linked to the game perspective. When a game presents a first-person approach, the playable characters/avatars are often omitted—they are there, but since the player is seeing the game world through their eyes, the actual characters are not seen. However, third-person point-of-view (POV) games generally allow the player to control a specific character/avatar that they can keep track of at all times [35,37].
Presence of a story (yes/no)	Whether the game play invites the player to follow a story.
Type of scenery/in-game environment (realistic/fantasy/simple)	Refers to the aesthetics of the background image/3D setting used in the game. Realistic sceneries depict situations, locations, etc, that can be found in the real world. Fantasy determines that the scenery represents locations that are not an imitation of direct reality. Simple environments denote that there is no actual scenery, and the game takes place on top of single-color backgrounds with no associated imagery, often relying on geometric shapes, hence without any specific sense of aesthetic.
Level of immersion applied to the use of VR^d^ (immersive/nonimmersive)	Immersion can be described as the sensation the player experiences as being part of the virtual world promoted by the game, that is, the involving nature of game play [38,39]. In this specific context (applied to VR), nonimmersive VR denotes a system where the interaction between environment and player is achieved through the use of a mouse or a joystick, putting some distance between player and game, while immersive VR implies the use of tools that are connected to the human body (eg, head-mounted display) in order to interact with the game [40].

^a^GG: game genre.

^b^1PP: first-person perspective.

^c^3PP: third-person perspective.

^d^VR: virtual reality.

### Data Synthesis and Analysis

The screening process was completed by the authors. The papers were selected by title and abstract if they met the inclusion criteria. If so, full-text papers were obtained for closer inspection. Any disagreement concerning whether to include a specific study was discussed with all the authors.

### Assessment of Quality

To assess the quality of the papers, we used the code “Quality of the Studies” by Connolly et al [41]. Each of the 12 papers included in this review was given a quality score using a 3-point Likert scale (3=high; 2=medium; 1=low) based on 5 questions, for example, *How appropriate is the research design for addressing the question or subquestions of this review (with a higher weighting for inclusion of a control group)?*; see more in Connolly et al [41]. After scoring each paper, the mean score and mode were calculated (mean 13.42, SD 0.64; mode=12), with scores ranging between 12 and 15 points. Following Connolly et al [41], we used the mode as a cutoff point to determine which papers should be included in this study in order to increase the probability of more methodologically robust evidence. Thus, papers with a score of 12 or more were included.

## Results

### Paper Selection Process

**Figure 1:**
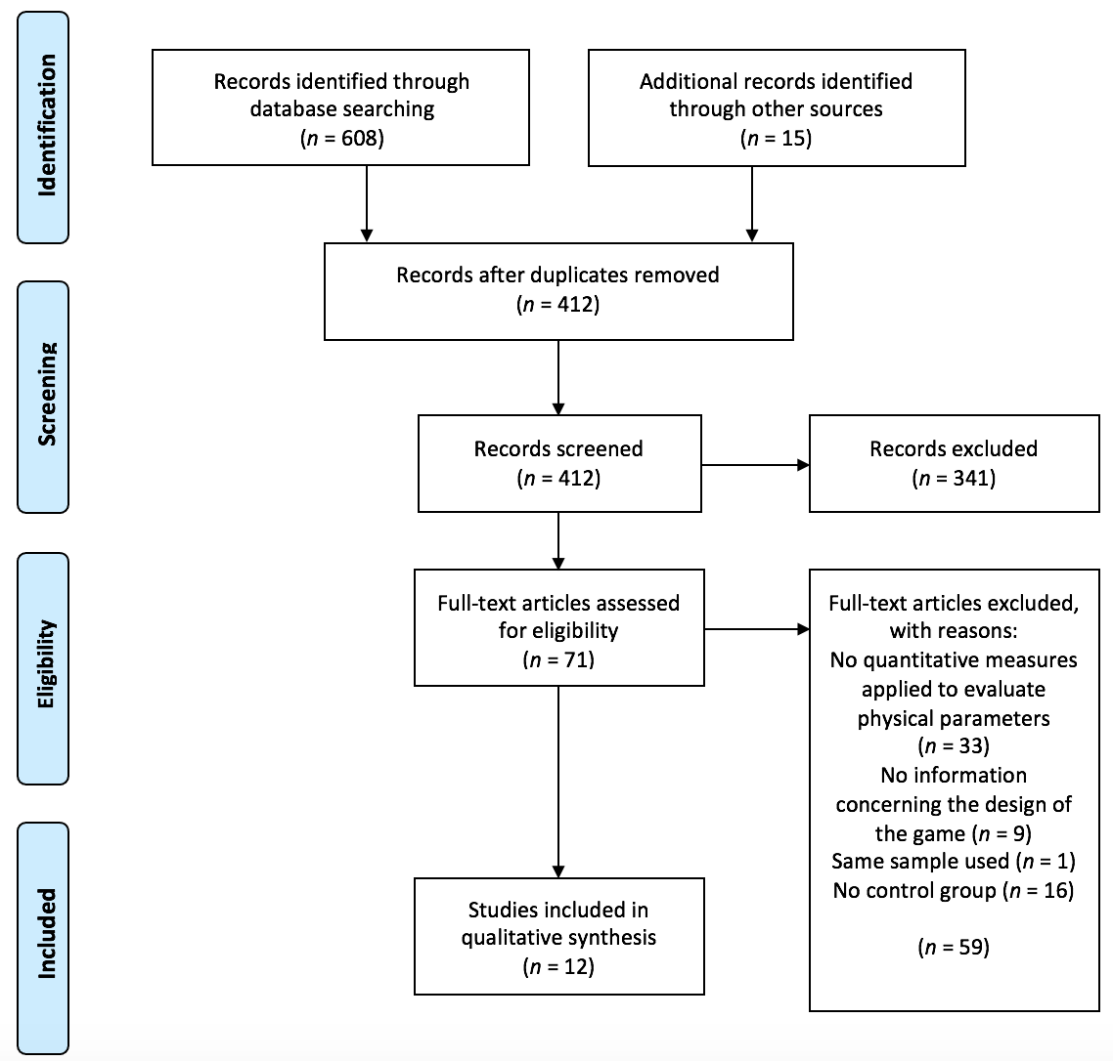
Article selection process for the systematic literature review.

The search conducted resulted in 623 papers, including 15 papers identified through recursive analysis (excluding duplicates). After removing duplicates, 412 papers with unique titles were identified and reviewed. After title screening, 202 papers were excluded. Another 139 were excluded after abstract review for not meeting the inclusion criteria. A comprehensive review of 71 full-text papers was conducted, 59 of which were excluded. As a result, 12 papers were selected for quality assessment. All of the 12 papers ranked 12 or more in the quality assessment (see Multimedia Appendix 1 for results) and were finally included in this systematic review (see Figure 1).

Regarding the organization of the studies, it is important to note that the cases analyzed in this review sometimes present more than one game mode (eg, sometimes the player can pick whether they want to play the game in 1PP or 3PP, and sometimes, they can play it solo or opt for a multiplayer function instead). When this happened, we selected the option that differentiated the game from other titles (eg, if the multiplayer function was available, even if not always used, the game was considered a multiplayer game). Additionally, in situations where COTS games were used, if there was any reference to the games not being played in full (eg, only certain features of the game were explored for therapy purposes), we solely considered the aspects that were, in fact, used in those studies. A table featuring a summary of the general characteristics of each study, displaying the pathology, rehabilitation goals, GDS, GG, GN, nature of intervention, evaluation measures, qualitative results, and quantitative results, is included in the supplementary materials of this paper (Multimedia Appendix 2).

### Studies and Sample Characteristics

Of the 12 papers, 7 were randomized controlled trials [11-15,17-21] and quasi-experimental controlled studies or pre /posttest designs [10,16]. A total of 512 participants (from all age groups) were included in this review, who had pathologies such as stroke (8 studies, 417 participants), cerebral palsy (1 study, 8 participants), and multiple sclerosis (2 studies, 46 participants); 1 study targeting the elderly (41 participants) was also included. The ages of the participants ranged from 18 to more than 85 years (estimated mean age ~59 years). For the stroke cases, the time after stroke went from 1 month up to over 2 years, although most of the patients in the sample were within the first semester after stroke. For the multiple sclerosis cases, the elapsed time since the disease was diagnosed went from 2 years up to over 20 years.

### Intervention Characteristics

All studies featured physical therapy sessions with durations varying from 25 minutes to 1 hour. The weekly frequency of the treatment was also flexible, ranging from twice a week to every day. The duration of the studies varied from 5 days to 10 weeks, and the number of sessions went from 5 up to 42.

### Measures and Clinical Assessment

To relate the clinical outcomes with the characteristics of the games used, it was necessary to create a classification for the clinical outcomes of the 12 studies presented in this review. Given that the studies were conducted with distinct experimental settings and patients, we proposed to classify the studies into two groups: studies that presented at least one significant improvement in their clinical measurements and studies that presented no significant improvement in any of the clinical measurements. All studies included in the review assessed the domain of motor, sensory, and functional functions, resorting to measures such as the 6-minute walk test (6MWT), the Wolf Motor Function Test (WMFT), the Box and Block Test (BBT), the Nine-Hole Peg Test (9HPT), the 10-meter walk test, the Tinetti test, the Timed Up & Go Test, the Dynamic Gait Index, the Berg Balance Scale (BBS), the Fugl-Meyer Assessment Upper Extremity Scale, Timed Chair Stands, the Purdue Pegboard Test (PPT), and the Action Research Arm Test. Additionally, some studies measured general health outcomes (eg, physical and psychological well-being, QoL, perception of health, cognition, emotion, communication, social participation, perception of pain) by using the Multiple Sclerosis Impact Scale, the EQ-5D visual analogue scale (EQ-VAS), the Short-Form 12 Health Survey, the Stroke Impact Scale (SIS), the visual analogic scale (VAS), and the Functional Independence Measure (FIM). These outcomes were occasionally presented as secondary outcomes [10,11,13,20]. Still other studies [11,20] assessed compliance or satisfaction outcomes (eg, the Client Satisfaction Questionnaire). The BBT (to measure unilateral gross manual dexterity), SIS, and VAS measures were used in 3 to 4 studies. In this review, interventions with games were essentially used for motor rehabilitation of the upper limbs [10-15,18,20,21].

### Study Scope, Clinical Improvements, and Session Characteristics

Of the 12 studies, 8 (66.6%) presented significant improvements in at least one of the clinical measurements.

Taking a closer look at studies that had significant outcomes, arm function improvements were found by Saposnik et al [21] in the WMFT and Jonsdottir et al [13] in the 9HTP and BBT. Bruno et al [17] found significant clinical improvements in terms of gait and balance in the BBS and Tinetti test, while meaningful improvements in terms of aerobic capacity and endurance were found by Bower et al [14] in the 6MWT, as well as transfers and mobility improvements in the FIM. In the study by Norouzi-Gheidari et al [15], improvements were found in terms of activities of daily living in the Motor Activity Log–Quality of Movement, while Popović et al [12] attained improvements in patient motivation in the intrinsic motivation inventory and therapy time tests and movement smoothness and speed in the modified drawing test. In the study by Cuesta-Gómez et al [11], improvements in terms of coordination, speed of movement, and fine and gross upper-limb dexterity were found in the 9HPT, BBT, and PPT, along with the grip strength measure. Bortone et al [16] found improvements in terms of kinesiological assessment.

In the 8 studies that showed clinical improvement, 4 (50%) aimed at poststroke upper-limb rehabilitation [12,14,15,21], 2 (25%) aimed at upper-limb rehabilitation of multiple sclerosis–derived conditions [11,13], 1 (12.5%) aimed at improvement of general body balance in patients of advanced age [17], and 1 aimed at rehabilitation therapy of children with cerebral palsy [16].

In addition to the previously presented 8 studies, the remaining 4 of the 12 studies (33.3%) presented no significant improvement in any of the clinical measurements [10,18-20]. Of these 4, 3 (75%) studies aimed at poststroke upper-limb rehabilitation [10,18,20] and 1 aimed at poststroke general body balance rehabilitation [19].

Concerning the sample and study characteristics of each study, in the 8 studies that showed clinical improvements, except for 2 studies (1 that accounted for 41 patients with a mean age of 81 years [17] and 1 that accounted for 8 patients with a mean age of 10.13 years [16]), all other 6 studies experimented on adult patients with an overall mean age of 59 years (min=42.2, max=61.3). The mean study duration was 3.9 weeks (min=5 days, max=10 weeks), with the session frequency varying from 2 to 5 sessions per week and the session duration ranging from 25 minutes to 1 hour. Studies that did not show clinical improvements (4 of 12, 33.3%) experimented on adult patients with an overall age ranging from 33 to 81 years. The mean study duration was 3.67 weeks (min=2 weeks, max=6 weeks), with the session frequency varying from 3 to 7 sessions per week and the session duration ranging from 45 minutes to 1 hour.

### Game Genre

Of the 8 studies that presented clinical improvements in at least one of the measures, 6 (75%) studies presented their games as being from the casual game genre [11-14,16,17]; 1 (12.5%) of those studies combined the casual game genre with the simulation and exergaming genres [13]. The remaining 2 (25%) studies presented their games as being from the sports genre combined with the simulation genre [15,21].

All the 4 studies that did not show any sort of significant clinical improvement presented their games as being from the sports genre; 1 study combined sports with the simulation genre [10], 1 with simulation and casual games genres [18], and 1 with the exergaming genre [19].

### Game Nature

Of the 8 studies that showed clinical improvements, regarding the game player’s perspective, 1 (25%) study [17] presented a 3PP game, while all others opted for 1PP games, 3 of which also presented a playable character [14,15,21]. All 8 studies opted for single-player games, and with exception of 1 game [16], which used a fully immersive VR strategy, all others opted for nonimmersive VR. The visual style and type of scenery (or game aesthetics) of the games were fairly distributed among the 3 possible subcategories, with 2 (25%) studies describing their games’ aesthetics as simple [11,15], 3 (37.5%) as real [12,13,21], and 3 as fantasy [14,16,17].

Of the 4 studies that did not show any sort of significant clinical improvement, regarding the game player’s perspective, with the exception of 1 (25%) study that presented a 1PP game [10], all others (75%) opted for 3PP games [18-20]. All 4 (100%) studies presented a playable character. In addition, 3 (75%) studies opted for single-player games, while 1 opted for a multiplayer approach [10]. With the exception of 1 game [10], which used a fully immersive VR strategy, all others opted for nonimmersive VR. The visual style and type of scenery of the games tended toward real aesthetics in 3 (75%) studies, with 1 (25%) study describing the employed game as having fantasy-type scenery [10].

Of the 12 games analyzed, none invited the player to follow a story during the playthrough.

### Game Development Strategy

Of the 8 studies that showed clinical improvements, 7 (87.5%) used custom-made games [11-17], while 1 (12.5%) opted for a COTS game [21]. Of the 4 studies that did not attain any significant clinical improvement, 1 (25%) used a custom-made game [10], while 3 (75%) opted for COTS games [18-20].

## Discussion

### Principal Findings

Although video games can belong to several different GGs, this review suggests that the casual genre seems to be linked to a higher chance of obtaining significant clinical outcomes (eg, movement speed, upper-limb function and dexterity, gait, balance) when the game is used for physical rehabilitation. From a GN perspective, games using 1PP, played in single-player mode and using nonimmersive VR, seem to be linked to positive clinical outcomes (eg, coordination, movement speed, upper-limb function, gait, balance, motivation). It is also important to mention that 1PP games can frequently be associated with the use of nonvisible playable characters, an aspect that was also linked to studies reporting significant clinical outcomes. Concerning the type of scenery/in-game environment, since the results were well distributed throughout the different possibilities (simple, real, fantasy), it was not possible to discern whether a video game’s visual aesthetic is directly linked to significant clinical outcomes. Although it is possible to attempt and draw conclusions in terms of game perspective, game-play mode, and level of immersion applied to the use of VR, it is not possible to conjecture with regard to how the presence of a story might influence the propitiousness for obtaining significant clinical improvements, considering no SGs abridged in this review featured one in their design. Additionally, this review shows that although both COTS and custom-made SGs can be used for physical rehabilitation, a higher percentage of the custom-made SG studies presented significant clinical results linked to patient improvement. Such findings can prove useful for health care professionals by aiding their process of choice upon needing to select a video game approach as a physical rehabilitation therapy method, which, in turn, can potentially result in a better experience for patients, as well as positive clinical outcomes.

### Game Genre

With regard to the GG and its relationship with clinical outcomes, the genre that showed the best results was casual games. As explored previously, a casual game can be described as any sort of video game that requires the player to complete single and simple tasks that are generally not linked to stories or any other form of longer game play. In addition, it does not require any previous knowledge of or background and experience in playing video games—the player learns how to play right away, and once the mini-game is completed, the playthrough of the game is finished. Casual games seem to be popular in rehabilitation as they include simple game-play types, such as drag-and-drop (the player is expected to simulate the act of grabbing a virtual object—using the mouse cursor, for example—and then dragging it across the screen to a specific location to trigger an action or outcome) or point-and-click (games centered around the action of moving the cursor to a specific point on the screen and then clicking or pressing a button to trigger an action) [42]. Some of the casual games found in this review involved the virtual playout of quotidian situations or tasks—a strategy that has already been demonstrated to achieve positive clinical outcomes [20].

In addition to answering rehabilitation needs, another interesting factor behind casual games is the development time. Being rather simple in terms of game play and short in terms of game length—the experience they offer aims to be simple and easy to understand, while still perceived as entertaining—when compared to games of other genres, casual games take a considerably lower amount of time to be designed, developed, and presented to consumers, which allows for thinner budgets without sacrificing game or aesthetic design quality [43]. This answers the premise of the minimum viable product (MVP), which refers to a product version that is used to collect validated learning from potential customers and users with the minimum amount of development effort, therefore saving time and cost resources [44].

### Game Nature

With regard to the GN, aspects such as game perspective, game-play mode, the presence of a story, and the presence of playable characters can highly influence the gaming experience, from the levels of motivation to the levels of experienced immersion upon game play [45,46].

In terms of physical rehabilitation, previous studies have proved that opting for 1PP enhances the sense of embodiment experienced by the user during playtime, therefore permitting better clinical outcomes and results [47,48]. This result is also supported by the cases analyzed in this review, which shows that the majority of the 1PP games portray positive clinical outcomes. This particular perspective is often found in the casual game genre, which matches the previous findings concerning GGs. Additionally, when games do not feature an option that allows the player to toggle between 1PP and 3PP, 1PP games are often linked to the absence of visible avatars/playable characters [49] (also commonly found in casual games), which also appears to be related to positive clinical outcomes, which is another aspect that favors casual games over other video GGs in this specific context. With this in mind, if we take another look at the concept of MVP, and since the use of 1PP implies that the player character/avatar is not visible, it is relevant to note that this works in favor of reducing production costs; if the playable character is not visible, there is no need to create human-like avatars or human-like movement animations.

Between single-player and multiplayer games, single-player titles were dominant. Moreover, the single study that used a multiplayer game did not present any significant clinical outcome. In addition, nonimmersive VR was preferred over immersive VR. This can possibly be attributed to the problems (such as motion sickness) that are still prevalent in this sort of approach, mostly related to a high degree of undesired latency [50,51], and also to the year when the studies took place. Immersive VR is only becoming something rather ordinary now, while nonimmersive VR video games have already been around (commercially) for decades.

In terms of the type of environment, there was no clear relationship between the type of aesthetics used in the games and the clinical outcomes of the studies. Although in SGs targeting education, it has been proved that a fantasy setting seems to attain better learning outcomes [52], this review found no clear evidence of 1 type of setting working better than another, considering that all possible categories (real, simple, fantasy) found in the games analyzed in this review were displayed evenly in the significant-positive-outcome group.

This review does not allow any specific conclusions on how the presence/absence of a story can influence the significance of outcomes, considering that no game was said to have a story that the player must follow during the game’s playthrough. This finding is curious, considering that when users buy games for their own entertainment without any serious purposes in mind, a large chunk of these consumers pick the games according to whether the video game’s plot “strikes their fancy.” In this sense, and even if games belonging to, for example, the casual genre, do not necessarily need a story to still be perceived as fun, every game can benefit from a good, gripping, and well-written story. With this in mind, and if the presence of a story plays such a big role in terms of play/game enjoyment, it comes across as crucial to look at it more closely, even when the game’s objective is targeting physical rehabilitation [53]. Thus, a larger number of cases with video games featuring stories would be needed to be able to discern their relevance in terms of therapeutic functionality and motivation/compliance enhancement.

### Game Development Strategy

The custom-made games showcased in this review presented significant clinical outcomes, making them undoubtedly promising as an approach to physical rehabilitation. Additionally, the obtained clinical outcomes also proved that custom-made SGs can be as functional as traditional rehabilitation approaches [11-17].

COTS games did not appear to result in any significant clinical improvements among the participants, although they are often perceived as fun by the users. This can be explained because COTS games were originally designed not as a therapeutic tool but as a form of entertainment: they are expected to be perceived as fun by their players, and their main purpose is to entertain. This may also play a role in how patients perceive the form of treatment they are offered, considering that a large number of the population knows and associates COTS franchises (Nintendo, for example) to a form of entertainment, or even a toy, and not something potentially clinically beneficial [54]. When studies resort to COTS games, they use Nintendo Wii games. These require the player to move around and, therefore, execute the needed motions for rehabilitation. Moreover, it might be potentially interesting to observe that some custom-made games presented in these papers were developed for Microsoft Kinect. Nevertheless, the differences in terms of how movement is captured and tracked between Nintendo Wii (tracking of the wireless joystick) and Microsoft Kinect (tracking of the full body) should not be disregarded. The Nintendo Wii joystick (wiimote) makes it easy for the player to cheat the necessary movement, since the device does not register the exact position of the player but just where the remote is located [4]. This does not happen in Microsoft Kinect, since the entire body is constantly tracked, not just the spatial position of the joystick. From this analysis, we can argue that the body movement and gestures performed while playing COTS games are only an approximation of the actual therapeutic movement required by clinical guidelines—hence, the low clinical effectiveness when compared to custom-made games.

Despite the presence of significant clinical outcomes in custom-made SGs, it has been highlighted that there is a need to make SGs more fun to further enhance motivation. Jonsdottir et al [13] pointed out that patients perceive more improvements when playing Wii games (COTS) than when playing games specifically designed for therapy (even if, clinically, that did not turn out to be the case). This opens up the possibility to go beyond the basic notion of custom+-made games and develop the final product to the taste and preference of the patient.

The concept of taking into account patient interests and introducing them as part of the game design elements is an idea that has already been used [55]. Moreover, this will allow custom-made games to conceptually grow closer to COTS games by providing their players with content that is of their interest, stimulating feelings of fun and entertainment that are typically associated with the experience of playing COTS video games.

Likewise, knowing that custom-made casual games can attain the same type of significant outcomes as traditional therapy, it becomes particularly advantageous to look at this approach as a way of monetizing physical therapy, while providing better access to rehabilitation to a larger number of patients. Since a single game can answer the needs of many patients with the same kind of disability, the time lapsed from the moment the investment is made until it being repaid in full is considerably short: SGs are expected to get a high return on investment [54].

However, if we want to take a look at how COTS games can potentially thrive and cross over with the GN elements previously analyzed, it is important to think about the avatar customization options often found in COTS games. When a video game uses avatars that aim to represent the player, it is important that the user identify with them. COTS games already try to tackle this issue by frequently offering avatar customization options, allowing the user to design their character according to their preferences in order to ease the process of identification with the avatar, create empathy, and promote a sense of embodiment [47,48].

It is also important to mention that although using COTS games for physical rehabilitation may, at first, seem like a great way to avoid the production costs of developing and deploying custom-made SGs, COTS games do not prove to be as effective as the games purposefully developed to be used in therapy, therefore making the investment in custom-made SGs worth it if it they are bound to result in better health outcomes for their users.

### Limitations

The main limitation of the present review has to do with the possible lack of accuracy in placing the games described in each study inside a specific genre, since, occasionally, the data provided in the papers included in this review concerning the design/game play of each game were scarce, making it hard to clearly pinpoint the exact genre of the game. Additionally, since this review only found and analyzed 12 relevant papers, which, in turn, only featured 4 distinct GGs, there is no way of knowing whether casual games are indeed the best approach, since the largest slice of the GG panoply was not represented in this review. The same happened with some of the GN features, such as the presence of a narrative, the game-play mode, and the type of scenery/in-game environment—with the information found in the papers included in this review, it is not possible to pinpoint any conclusive findings.

### Conclusions

This review had the objective to provide some insight into what kind of video games seem to work better for physical rehabilitation in terms of GG and GN characteristics. The review and analysis presented here were able to infer important discussion and conclusions on this topic, informed by actual quantitative and qualitative clinical results. As looking at rehabilitation from an SG design point of view is a perspective that is still little explored or formalized, to achieve more robust conclusions, it is necessary for the community to also present and discuss in detail the game design–related aspects of clinical studies.

Nevertheless, this review allows us to conclude that when speaking about SG development strategies, custom-made titles are able to attain better, significant clinical outcomes compared with COTS games since they are designed with therapy-specific movements in mind, therefore not opening the doors to cheating or being perceived as a form of entertainment or a toy.

Of the genres featured in the papers included in this review, casual games obtained the best clinical outcomes in terms of significance (6 of 8, 75%, attained significant results). If we delve even deeper and take a look at how GN features may influence outcomes, both 1PP video games as well as video games that did not feature a visible playable character were behind in significant clinical outcomes. As previously explored, these two features generally go hand-in-hand: if a game is played from the user’s point of view, then it is to be expected that the player does not “see” the character/avatar they are controlling. Likewise, this contributes toward reducing production costs and development time, making this approach feasible not only from a clinical outcomes’ perspective but also from an economic point of view.

## Appendix

Multimedia Appendix 1 Quality assessment results.

Multimedia Appendix 2 Summary of included studies’ information.

## Abbreviations

1PP: first-person perspective
3PP: third-person perspective
6MWT: 6-minute walk test
9HPT: Nine-Hole Peg Test
BBS: Berg Balance Scale
BBT: Box and Block Test
COTS: commercial off-the-shelf
FIM: functional independence measure
MVP: minimum viable product
PPT: Purdue Pegboard Test
PRISMA: Preferred Reporting Items for Systematic Reviews and Meta-Analyses
QoL: quality of life
RTT: received therapy time
SIS: Stroke Impact Scale
VAS: visual analogic scale
VR: virtual reality
WMFT: Wolf Motor Function Test
