# Supplementary material for: Serious Game Design and Clinical Improvement in Physical Rehabilitation: Systematic Review
Source: JMIR Serious Games. 2021 Sep 23;9(3):e20066. doi: 10.2196/20066 (PMC8498892; doi:10.2196/20066)
Supplement: Multimedia Appendix 1 [file games_v9i3e20066_app1.docx]

Multimedia Appendix I

Quality assessment results.

| **Reference** | **1. How appropriate is the research design for addressing the question, or sub-questions of this review (higher weighting for inclusion of a control group)?** | **2. How appropriate are the methods & analysis?** | **3. How generalisable are the findings of this study to the target population with respect to the size and representativeness of sample. To what extent would the findings be relevant across age groups (14þ), gender, ethnicity, etc?** | **4. How relevant is the particular focus of the study (including conceptual focus, context, sample and measures) for addressing the question or sub-questions of this review?** | **5. To what extent can the study findings be trusted in answering the study question(s)?** | **total score (5-15)** |
| --- | --- | --- | --- | --- | --- | --- |
| (Saposnik et al., 2010) | 3 | 3 | 2 | 3 | 3 | 14 |
| (Triandafilou et al., 2018) | 2 | 2 | 2 | 3 | 3 | 12 |
| (Adie et al., 2017) | 3 | 3 | 3 | 3 | 3 | 15 |
| (Saposnik et al., 2016) | 3 | 3 | 2 | 3 | 3 | 14 |
| (Cuesta-Gómez et al., 2020) | 3 | 3 | 2 | 3 | 3 | 14 |
| (Popovic, KostiÄ‡, RodiÄ‡, & KonstantinoviÄ‡, 2014) | 2 | 2 | 2 | 3 | 3 | 12 |
| (Jonsdottir et al., 2018) | 2 | 2 | 2 | 3 | 3 | 12 |
| (Bower et al., 2015) | 2 | 2 | 2 | 3 | 3 | 12 |
| (Norouzi-Gheidari et al., 2020) | 3 | 3 | 1 | 3 | 3 | 13 |
| (Bortone et al., 2018) | 3 | 3 | 1 | 3 | 3 | 13 |
| (Bruno et al., 2017) | 2 | 2 | 2 | 3 | 3 | 12 |
| (Deutsch, Guarrera-Bowlby, & Kafri, 2017) | 3 | 3 | 1 | 3 | 3 | 13 |
