# Supplementary material for: Serious Game Design and Clinical Improvement in Physical Rehabilitation: Systematic Review
Source: JMIR Serious Games. 2021 Sep 23;9(3):e20066. doi: 10.2196/20066 (PMC8498892; doi:10.2196/20066)
Supplement: Multimedia Appendix 2 [file games_v9i3e20066_app2.docx]

Multimedia Appendix II

Summary of included studies’ information.

| **Reference** | **Game Development Strategy and Genre** | **Pathology + Rehab Goals** | **Nature of Game (gameplay, design ,…)** | **Nature of Intervention** | **Evaluation Measures** | **Qualitative Results** | **Significance of Results (Measures)** |
| --- | --- | --- | --- | --- | --- | --- | --- |
| Triandafilou et al., 2018 | Custom Made  —Simulation;  —Sports; | Stroke – upper limb rehabilitation; | — Game Perspective: 1^st^ person POV;  — Presence of Narrative: Not present.  — Number of players: multiplayer;  — Presence of Playable Characters: Yes;  — Environment: Fantasy setting;  — VR type: Immersive. | — Pilot Study  — Testing design with 2 control groups;  — 15 stroke patients (10 male, 5 female, ages between 33 – 81 years old).  — Study duration: 3 weeks;  — Total number of sessions: 9;  — Session duration: 1 hour;  — 3 sessions/week; | — VERGE survey (questionnaire)  — Weekly survey (including VERGE) at the end of each therapy week.  — Reach distance;  — Hand elevation;  — Arm displacement; | — 13 out of 15 participants found the therapy very or extremely productive. 14 participants found themselves satisfied or very satisfied.  — Participants largely enjoyed playing with a partner (the presence of another user increased motivation).  — The patients still show more interest in traditional approaches. | Non- significant. |
| Bower et al., 2015 | Custom Made:  —Casual Games | Stroke – upper limb and torso rehabilitation; | — Game Perspective: 1^st^ person POV;  — Presence of Narrative: Not present.  — Number of players: single player;  — Presence of Playable Characters: Yes;  — Environment: Fantasy setting;  — VR type: Non-immersive. | *Study divided in 2 phases:*  **Phase 1**: initial feasibility testing; 40 individuals (mean age 63 years) were randomly assigned to trial one of the four available games during a single session; Participants played 1 of the 4 games (randomly selected) in a single session, under professional supervision;  **Phase 2:**  —Pilot randomized controlled trial;  — Features a control group;  —16 participants (mean age 61 years) from phase 1 were recruited;  — Study duration: 4 weeks;  — Total number of sessions: 8;  — Session duration: 40 min;  — 2 sessions/week; | — Several functional outcomes were also assessed (at baseline and 4 weeks after starting phase 2):  — FIM;  — Motor Assessment Scale;  — Functional Reach;  — Step Test;  — 6MWT. | — In terms of enjoyment, the participants claimed that they felt the games were a fun and fresh way to participate in therapy.  — The intervention group improved significantly in several of the measured outcomes. | Significant:  — 6MWT;  — FIM; |
| Saposnik et al., 2010 | COTS:  — Sports;  — Simulation; | Stroke Rehabilitation – upper limb rehabilitation. | — Game Perspective: 1^st^ person POV (Cooking Mama); 3^rd^ person POV (Wii Sports)  — Presence of Narrative: Not present.  — Number of players: multiplayer (although possibly used as a single player game);  — Presence of Playable Characters: Yes;  — Environment: Realistic setting  — VR type: Non-immersive. | — Canada  — 22 stroke patients (mean age was 61.3).  — Features a control group that engages in Recreational Therapy playing cards, bingo or “Jenga”.  — Study duration: 14 days;  — Total number of sessions: 8;  — Session duration: 1 hour; | Measured outcomes (evaluated 4 weeks after intervention):  — Primary safety and feasibility;  — The level of effort necessary to complete the games;  — Wolf Motor Function Test (WMFT);  — Box and Block Test (BBT);  — Stroke Impact Scale (SIS).  — Grip Strength. | — The participants of the VRWii group showed significant improvements in motor arm function. | Significant:  —WMFT; |
| Jonsdottir et al., 2018 | COTS:  — Health & Wellness/Fitness /Exergaming;  Custom Made: — Simulation;  — Casual Games; | Multiple Sclerosis – upper limb rehabilitation. | No specification of the Wii games used, other than the fact that they were Exergames.  Rehab@Home:  — Game Perspective: 1^st^ person POV;  — Presence of Narrative: Not present.  — Number of players: single player;  — Presence of Playable Characters: Not present;  — Environment: Realistic setting  — VR type: Non-immersive. | — 16 patients suffering from multiple sclerosis (mean age = 56.8);  — 10 participants used Rehab@Home – experimental group; 6 participants used Nintendo Wii – control group;  — Study duration: 4 weeks;  — Total number of sessions: 12;  — Session duration: 40 min;  — 4-5 sessions/week; | — Primary outcomes:  9 Hole Peg Test (9HPT); Box and Block Test (BBT);  — Secondary outcomes:  EQ-5D visual analogue scale (EQ-VAS); SF-12; | — Positively perceived in terms of user experience and motivation.  —The serious games group showed clinically significant improvements in arm function.  — The Wii group showed no significant improvements on any of the tests.  — Despite this fact, only the Wii group perceived themselves as having improved their health. | Significant:  — 9HPT;  — BBT;  — EQ-5D-VAS; |
| Norouzi-Gheidari et al., 2020 | Custom Made: — Sports;  — Simulation; | Stroke Rehabilitation – upper limb rehabilitation; | **Jintronix:**  — Game Perspective: 1^st^ person POV;  — Presence of Narrative: Not present.  — Number of players: single player;  — Presence of Playable Characters: Yes;  — Environment: Simple setting  — VR type: Non-immersive. | — Single-blind pilot randomized clinical control trial;  — 9 participants (5 male, 4 female, mean age 42.2) were assigned to the intervention group and 9 subjects (5 male, 4 female, mean age 57.6) were assigned to the control group;  — Study duration: 4 weeks;  — Total number of sessions: 8+;  — Session duration: ~ 44 min;  — 2 sessions/week; | — MAL-QOM;  — SIS;  — FMA-UE;  — BBT;  (Borg Scale and VAS to monitor fatigue and pain) | — Some patients maintained gains in the follow-up period.  —Feasible to use VR exergaming as an adjunct to traditional therapy. | Significant:  — MAL-QOM; |
| Saposnik et al., 2016 | COTS:  — Wii Sports;  — Simulation;  — Mini-games; | Stroke Rehabilitation – upper limb rehabilitation. | **Wii Sports and Game Party 3:**  — Game Perspective: 3^rd^ person POV;  — Presence of Narrative: Not present.  — Number of players: multiplayer (although possibly used as a single player game);  — Presence of Playable Characters: Yes;  — Environment: Realistic setting  — VR type: Non-immersive. | — Controlled, single-blind, parallel-group, randomized trial.  — 141 patients ( 94 males, 47 females) were selected but only 121 completed the final assessment (59 in the VRWii group and 62 in recreational therapy);  — Study duration: 2 weeks;  — Total number of sessions: 10;  — Session duration: 1 hour  — 5 sessions/week; | *Primary:*  — WMFT  *Secondary:*  — BBT  — Barthel Index  — FIM  — SIS  — Grip Strength | — The type of task in post-stroke motor rehabilitation might be less relevant, as long as it is intensive and task-specific.  — No significant difference in terms of clinical outcomes was found. | Non-significant. |
| Bortone et al., 2018 | Custom Made:  — Casual Games | Rehabilitation Therapy of children with Cerebral Palsy (CP) and/or Developmental Dyspraxia (DD) | **Moneybox and Marble Labyrinth:**  — Game Perspective:1^st^ person POV;  — Presence of Narrative: Not present;  — Number of players: single player;  — Presence of Playable Characters: Not present;  — Environment: Fantasy setting  — VR type: Immersive. | — Pre-post testing design with control group;  — Three groups: ill children (8 participants, mean age 10.13, CP and DD patients);  — Healthy children (TD) with regular development group (8 children, mean age 13.38); Healthy adult (AD) group (4 adults, mean age 26.75); | — Movement Speed:   - Movement time; - Mean velocity;   — Movement accuracy:   - RMSE - Target error. | — VR enhances motivation, making it a potentially important rehabilitation tool for children.  — Results showed to be consistent with the participants’ motor skills. | Significant:  — Kinesiological Assessment; |
| Deutsch, Guarrera-Bowlby, & Kafri, 2017 | COTS:  — Sports;  — Health & Wellness/Fitness /Exergaming; | Stroke – Balance; | — Game Perspective: 3^rd^ person POV;  — Presence of Narrative: Not present;  — Number of players: single player;  — Presence of Playable Characters: Yes;  — Environment: Realistic setting;  — VR type: Non-immersive. | — Double-blind randomized controlled pilot study;  — Pre-post testing design with control group;  —Two groups: Standard of Care (5 participants, 4 male, 1 female, mean age 49) and Video-game based balance group (5 participants, 4 male, 1 female, mean age 53);  — Study duration: 4 weeks;  — Total number of sessions: 12;  — Session duration: 1 hour;  — 3 sessions/week; | — DGI  — Gait Speed  — ABC  — COPM performance  — COPM satisfaction | — Both groups improved.  — This study believes that the customization offered by COTS may help overcome some of the limitations of using COTS. | Non-significant. |
| Bruno et al., 2017 | Custom Made:  — Casual Games; | General Elderly – Balance; | The Pirate, The Wipe Out, The Flight Simulator, Hit the Box, Drop the Ball.  — Game Perspective: 3^rd^ person POV (only The Wipe Out is in 1^st^ person POV);  — Presence of Narrative: Not present;  — Number of players: single player;  — Presence of Playable Characters: Not present;  — Environment: Fantasy setting;  — VR type: N/A; | — Preliminary study  — Pre-post testing design with control group.  — 41 patients (mean age 81 years old) allocated into 3 different groups:   - Combination of Serious Games + PT; - Combination of PT + dual task training; - Standard PT and another sessions of PT of the same duration of the double task training and SG.   — 9 patients left the study during the protocol;  — Study duration: 5 days;  — Total number of sessions: 5;  — Session duration: 30 min;  — 5 sessions/week; | — Tinetti;  — BBS;  — TUGT;  — TCS;  — 10m DT; | — SG can be added in the conventional treatment of hospitalized elderly patients.  — SG contribute to maintain the motivation of the patients while bringing novelty into the physical rehabilitation process. | Significant:  — BBS;  — Tinetti; |
| Cuesta-Gómez et al., 2020 | Custom Made:  — Casual Game | Multiple Sclerosis (MS) – Upper limb rehabilitation | Piano Game, Reach Game, Sequence Game, Grasp Game, Pinch Game, Flip Game:  — Game Perspective: 1^st^ person POV;  — Presence of Narrative: Not present;  — Number of players: single player;  — Presence of Playable Characters: Not present;  — Environment: Simple setting;  — VR type: Non-immersive. | — Spain  — Single-blinded randomized controlled trial;  — Pre-post testing design with control group;  — 30 patients (mean age 46.66, 12 male, 18 female) - experimental group: 16 patients (7 male, 9 female, mean age 49.86); control group: 14 patients (5 male, 9 female, mean age 42.66)  — Study duration: 10 weeks;  — Total number of sessions: 20;  — Session duration: 1 hour;  — 2 sessions/week; | — Grip Strength (Jamar)  — BBT  — PPT  — 9HPT  — FFS  — MSIS-29  — CSQ-8  — Attendance rate | — The compliance to the interventions was excellent.  — Specific SG are necessary for upper limb rehabilitation in patients with MS.  — There needs to be choice and space for personalization when it comes to SG applied to upper limb rehabilitation of MS patients.  — The patients were satisfied with the technology that was implemented. | Significant:  — 9HPT;  — BBT;  — Grip Strength;  — PPT; |
| Adie et al., 2017 | COTS:  — Sports; | Stroke – Upper limb rehabilitation; | — Game Perspective: 3^rd^ person POV;  — Presence of Narrative: Not present;  — Number of players: multiplayer;  — Presence of Playable Characters: Yes;  — Environment: Realistic setting;  — VR type: Non-immersive. | — Randomized controlled trial  — Pre-post testing design with control group;  — 240 participants were recruited but only 209 completed the study.  — Intervention group: 101 participants; Control group: 108 participants. Mean age 67.3; 144 were women.  — Study duration: 6 weeks;  — Total number of sessions: 42;  — Session duration: 45 min;  — 7 sessions/week; | — ARAT;  — Stroke Impact Questionnaire;  — COPM (Satisfaction and Performance);  — MAL;  — EQ 5D 3L VAS; | — Wii was not superior to traditional arm exercises.  — Well tolerated, but more expensive. | Non-significant. |
| Popović, Kostić, Rodić, & Konstantinović, 2014 | Custom Made:  — Casual Game; | Stroke: Upper Limb rehabilitation; | — Game Perspective: 1^st^ person POV;  — Presence of Narrative: Not present;  — Number of players: single player;  — Presence of Playable Characters: Not present;  — Environment: Realistic setting;  — VR type: N/A; | — Single blinded study.  — Pre-post testing design with control group;  — 20 participants allocated to 2 groups: experimental (FME) (10 participants, mean age 58) and control (NFE) (10 participants, mean age 57).  — Study duration: 3 weeks;  — Total number of sessions: 15;  — Session duration: 25 min;  — 5 sessions/week; | — mDT;  — RTT;  — IMI; | — High levels of motivation were found.  — Therapy endurance improved over the course of the intervention.  — Participants suggested changing the game task into a more interesting activity. | Significant:  — IMI;  — mDT;  — RTT; |

**Measures used (acronyms) and their meanings:**

- 10-MWT: 10-meter Walk Test.
- 6MWT: 6-minute walk test;
- 9HPT: Nine Hole Peg Test
- ABC: Activities-Specific Balance Confidence
- ARAT: Action Research Arm Test
- BBS: Berg Balance Scale
- BBT: Box and Block test;
- COPM: Canadian Occupational Performance Measure
- CSQ-8: The Client Satisfaction Questionnaire
- DGI: Dynamic Gait Index
- FIM: Functional Independence Measure
- FMA-UE: Fugl-Meyer Assessment-Upper Extremity Scale;
- FRT: Functional Reach Test
- FSS: Fatigue Severity Scale
- IMI: Intrinsic Motivation Inventory.
- MAL-QOM: Motor Activity Log-Quality of Movement;
- MAL: Motor Activity Log
- mDT: The Modified Drawing Test
- MSIS-29: Multiple Sclerosis Impact Scale
- PPT: The Purdue Pegboard Test
- RTT: Received Therapy Time;
- SF-12: Short-Form 12 Health Survey
- SIS: Stroke Impact Scale;
- TCS: Timed Chair Stands
- TTS: Tinetti Balance Test
- TUGT: Timed up and Go Test
- VAS: Visual Analogic Scale
- WMFT: Wolf Motor Function Test

**References/Bibliography:**

Adie, K., Schofield, C., Berrow, M., Wingham, J., Humfryes, J., Pritchard, C., … Allison, R. (2017). Does the use of Nintendo Wii SportsTM improve arm function? Trial of WiiTM in Stroke: A randomized controlled trial and economics analysis. *Clinical Rehabilitation*, *31*(2), 173–185. https://doi.org/10.1177/0269215516637893

Bortone, I., Leonardis, D., Mastronicola, N., Crecchi, A., Bonfiglio, L., Procopio, C., … Frisoli, A. (2018). *Wearable Haptics and Immersive Virtual Reality Rehabilitation Training in Children With Neuromotor Impairments*. *26*(7), 1469–1478.

Bower, K. J., Louie, J., Landesrocha, Y., Seedy, P., Gorelik, A., & Bernhardt, J. (2015). Clinical feasibility of interactive motion-controlled games for stroke rehabilitation. *Journal of NeuroEngineering and Rehabilitation*, *12*(1), 1–12. https://doi.org/10.1186/s12984-015-0057-x

Bruno, B., Melissa, V. V., Christophe, B., Sandra, D. B., Serge, V. S. J., Veronique, F., & Bart, J. (2017). A preliminary study of the integration of specially developed serious games in the treatment of hospitalized elderly patients. *International Conference on Virtual Rehabilitation, ICVR*, *2017*-*June*. https://doi.org/10.1109/ICVR.2017.8007508

Cuesta-Gómez, A., Sánchez-Herrera-Baeza, P., Oña-Simbaña, E. D., Martínez-Medina, A., Ortiz-Comino, C., Balaguer-Bernaldo-De-Quirós, C., … Cano-De-La-Cuerda, R. (2020). Effects of virtual reality associated with serious games for upper limb rehabilitation inpatients with multiple sclerosis: Randomized controlled trial. *Journal of NeuroEngineering and Rehabilitation*, *17*(1), 1–10. https://doi.org/10.1186/s12984-020-00718-x

Deutsch, J., Guarrera-Bowlby, P., & Kafri, M. (2017). Participation outcomes differed between video game-based balance training and standard of care training: Pilot clinical trial. *International Conference on Virtual Rehabilitation, ICVR*, *2017*-*June*. https://doi.org/10.1109/ICVR.2017.8007544

Jonsdottir, J., Bertoni, R., Lawo, M., Montesano, A., Bowman, T., & Gabrielli, S. (2018). Serious games for arm rehabilitation of persons with multiple sclerosis. A randomized controlled pilot study. *Multiple Sclerosis and Related Disorders*, *19*(August 2017), 25–29. https://doi.org/10.1016/j.msard.2017.10.010

Norouzi-Gheidari, N., Hernandez, A., Archambault, P. S., Higgins, J., Poissant, L., & Kairy, D. (2020). Feasibility, safety and efficacy of a virtual reality exergame system to supplement upper extremity rehabilitation post-stroke: A pilot randomized clinical trial and proof of principle. *International Journal of Environmental Research and Public Health*, *17*(1), 1–11. https://doi.org/10.3390/ijerph17010113

Popović, M. D., Kostić, M. D., Rodić, S. Z., & Konstantinović, L. M. (2014). Feedback-mediated upper extremities exercise: Increasing patient motivation in poststroke rehabilitation. *BioMed Research International*, *2014*. https://doi.org/10.1155/2014/520374

Saposnik, G., Cohen, L. G., Mamdani, M., Pooyania, S., Cheung, D., Shaw, J., … Outcomes, S. (2016). *Efficacy and safety of non-immersive virtual reality exercising in stroke rehabilitation (EVREST): a randomised, multicentre, single-blind, controlled trial*. *15*(10), 1019–1027. https://doi.org/10.1016/S1474-4422(16)30121-1.Efficacy

Saposnik, G., Teasell, R., Mamdani, M., Hall, J., McIlroy, W., Cheung, D., … Bayley, M. (2010). Effectiveness of virtual reality using wii gaming technology in stroke rehabilitation: A pilot randomized clinical trial and proof of principle. *Stroke*, *41*(7), 1477–1484. https://doi.org/10.1161/STROKEAHA.110.584979

Triandafilou, K. M., Tsoupikova, D., Barry, A. J., Thielbar, K. N., Stoykov, N., & Kamper, D. G. (2018). Development of a 3D, networked multi-user virtual reality environment for home therapy after stroke. *Journal of NeuroEngineering and Rehabilitation*, *15*(1), 1–13. https://doi.org/10.1186/s12984-018-0429-0
